# *JcDREB2*, a Physic Nut AP2/ERF Gene, Alters Plant Growth and Salinity Stress Responses in Transgenic Rice

**DOI:** 10.3389/fpls.2017.00306

**Published:** 2017-03-06

**Authors:** Yuehui Tang, Kun Liu, Ju Zhang, Xiaoli Li, Kedong Xu, Yi Zhang, Jing Qi, Deshui Yu, Jian Wang, Chengwei Li

**Affiliations:** ^1^Henan Key Laboratory of Crop Molecular Breeding and BioreactorZhoukou, China; ^2^Key Laboratory of Plant Genetics and Molecular Breeding, Zhoukou Normal UniversityZhoukou, China

**Keywords:** AP2/ERF transcription factor, physic nut (*Jatropha curcas* L.), salinity, gibberellin, rice

## Abstract

Transcription factors of the AP2/ERF family play important roles in plant growth, development, and responses to biotic and abiotic stresses. In this study, a physic nut AP2/ERF gene, *JcDREB2*, was functionally characterized. Real-time PCR analysis revealed that *JcDREB2* was expressed mainly in the leaf and could be induced by abscisic acid but suppressed by gibberellin (GA) and salt. Transient expression of a JcDREB2-YFP fusion protein in Arabidopsis protoplasts cells suggested that JcDREB2 is localized in the nucleus. Rice plants overexpressing *JcDREB2* exhibited dwarf and GA-deficient phenotypes with shorter shoots and roots than those of wild-type plants. The dwarfism phenotype could be rescued by the application of exogenous GA_3_. The expression levels of GA biosynthetic genes including *OsGA20ox1*, *OsGA20ox2*, *OsGA20ox4*, *OsGA3ox2, OsCPS1*, *OsKO2*, and *OsKAO* were significantly reduced in plants overexpressing *JcDREB2*. Overexpression of *JcDREB2* in rice increased sensitivity to salt stress. Increases in the expression levels of several salt-tolerance-related genes in response to salt stress were impaired in *JcDREB2*-overexpressing plants. These results demonstrated not only that *JcDREB2* influences GA metabolism, but also that it can participate in the regulation of the salt stress response in rice.

## Introduction

Abiotic stresses, such as high salinity and drought, adversely affect plant growth and agricultural productivity, resulting in serious losses in yield. To cope with these adverse conditions, plants have evolved various defense mechanisms enabling them to tolerate environmental stresses. Transcription factors are significant candidates for mediating plant responses to stress tolerance because of their roles as master regulators of many stress-responsive genes (Singh et al., 2002). In the past few years, many transcription factors belonging to the AP2/ERF, NAC, MYB, WRKY, bHLH, and NF-Y families have been characterized and shown to play significant roles in plant responses to biotic and abiotic stresses (Li et al., 2010; Jiang et al., 2014; Yan et al., 2014; Chen M. et al., 2015; Shao et al., 2015; Zhang et al., 2015; Hong et al., 2016; Tang et al., 2016).

The AP2/ERF transcription factors are defined by a conserved AP2/ERF domain which consists of about 60 to 70 amino acids, and they can be divided into four subfamilies, AP2, ERF, DREB, RAV and Soloist, depending on the number of AP2/ERF domains they contain and the presence or absence of other DNA binding domains (Sakuma et al., 2002; Wessler, 2005). Recently there has been increasing evidence that the AP2/ERF family proteins are involved in the responses of plants to biotic and abiotic stresses. For instance, DREB transcription factor proteins bind to the DRE/CRT (drought responsive/C-Repeat) *cis*-acting elements (core motif: G/ACCGAC) and control the expression of many stress-responsive genes (Shinozaki and Yamaguchi-Shinozaki, 2000). In rice and Arabidopsis, DREB transcriptional activators such as those encoded by *OsDREB2A*, *DREB2C*, and *AtDREB1A* have been isolated. *OsDREB2A* expression is induced by drought, low-temperature and salt stresses, and overexpression of *OsDREB2A* significantly affects salt tolerance by altering the accumulation of osmolytes, such as soluble sugars and free proline, in transgenic soybean (Zhang et al., 2012). Overexpression of *DREB2C* and *AtDREB1A* confers tolerance to, respectively, salt and drought in transgenic plants (Song et al., 2014; Wei et al., 2016). DREB-encoding genes in several other species, genes which include *VvDREB2A*, *SsDREB*, and *GmDREB1*, have been identified as playing significant parts in salinity tolerance through regulating the expression of stress-responsive genes and physiological processes (Jin et al., 2010; Zhang et al., 2014; Chen H. et al., 2015). DREB transcription factors are also involved in plant growth and development. Overexpression of *TINY*, a DREB subfamily gene, results in a dwarf phenotype in Arabidopsis under normal growth conditions (Sun et al., 2008). Although many DREB transcription factors have been extensively studied in various plants, further efforts are still needed to identify other novel DREB genes that are involved in plant development and stress responses.

Physic nut (*Jatropha curcas* L.) is a multipurpose perennial woody plant belonging to the Euphorbiaceae family. Its abilities to grow easily in barren soil and endure drought and salinity, its adaptation to a wide range of agro-climatic conditions, and its high seed oil content, mean that the physic nut has emerged as a promising source of biodiesel (Openshaw, 2000). Our previous study identified 119 putative AP2/ERF genes in the physic nut genome, and we observed that a gene (JCGZ_24071) which we designated *JcDREB2* was strongly repressed by salt stress (Tang et al., 2016). *JcDREB2* was therefore chosen for further functional analysis. In the present study, we demonstrate that *JcDREB2* encodes a transcription factor whose expression is regulated by salinity, gibberellic acid (GA_3_) and abscisic acid (ABA) treatments. Our results indicated that overexpression of *JcDREB2* in rice resulted in both a dwarf phenotype, via downregulation of key genes involved in GA biosynthesis, and increased sensitivity to salinity stress.

## Materials and Methods

### Plant Materials

*Jatropha curcas* seeds were collected from Guizhou province, China and planted on farm land in Guangzhou, Guangdong province, China. For analysis of *JcDREB2* gene expression in physic nut, roots, stem cortex, leaves, flowers, and seeds were sampled at 35 days after pollination and stored at -80°C until required for RNA isolation. For salinity treatment, plants at the six-leaf stage were irrigated with Hoagland solution plus 150 mM NaCl. For GA_3_ and ABA treatments, plants at the six-leaf stage were sprayed with 100 μM GA_3_, 100 μM ABA or distilled water (control), and the fourth leaves were collected after 1, 3, 6, and 12 h of GA_3_ and ABA stresses.

The *japonica* rice (*Oryza sativa* L.) cv. Zhonghua 11 (ZH11) was used as the wild type in this study. Seeds were germinated and cultured in soil in basins in a greenhouse under natural sunlight.

### Protein Sequence and Phylogenetic Analyses

All protein sequences of JcDREB2 orthologs were downloaded from GenBank^1^. ClustalX was used to analyze multiple sequence alignment (Thompson et al., 1997). The phylogenetic relationship of JcDREB2 orthologs was constructed, using MEGA 5 software, by the Neighbor–Joining method with 1000 bootstrap replicates (Tamura et al., 2011).

### Subcellular Localization

The full length coding domain sequence of *JcDREB2* (after removal of the termination codon) was amplified by RT-PCR from total RNA extracted from physic nut leaves using the primers given in Supplementary Table S1. The target sequence was cloned into the pSAT6-eYFP-N1 vector to construct the pSAT6-*JcDREB2*-eYFP fusion expression vector. The 35S::*JcDREB2*-YFP construct and a control (35S::YFP) plasmid were introduced into Arabidopsis protoplasts by the polyethylene glycol (PEG) mediated method. Next the transformed cells were incubated in the light for 16 h at 25°C. YFP fluorescence signal was detected by laser scanning confocal microscopy. Arabidopsis protoplasts was prepared according to Axelos (1992).

### Gene Cloning and Plant Transformation

Total RNA was isolated from the leaves of 14-day-old physic nut seedlings using TRIzol reagent (Invitrogen, Carlsbad, CA, USA) according to the manufacturer’s instructions, and first-strand cDNA was obtained following Tang et al. (2016). *JcDREB2* fragments including the complete coding sequences were amplified from physic nut by PCR with the primer pairs shown in Supplementary Table S1. The products were then cloned into the vector pMD 18-T (TaKaRa, Otsu, Japan) for use in sequencing and analysis. The target sequences were digested with the restriction enzymes *Sac*-I and *Xba*-I and then inserted into the corresponding restriction sites of the pCAMBIA1301 vector under the control of a CaMV35S promoter. Next, Agrobacterium harboring the constructs was used to transform and regenerate rice seedlings, as described by Chen et al. (2003). In total we obtained seven independent transgenic lines, and selected three single gene insertion homozygous transgenic lines for further study based on an approximately 3:1 segregation ratio being observed among T2 plants. The segregation ratios, which were confirmed by GUS staining, were 216:70, 198:63 and 237:76. T2 seeds were germinated and 80 roots were taken from 10-day-old seedlings of each line of transgenic rice for GUS staining. GUS expression was observed in all roots from each line of 10-day-old seedlings.

### Stress Treatments

After germination, rice seedlings were cultured in Yoshida’s culture solution (Yoshida et al., 1976) at 25°C under 16/8 h (light/dark) conditions in a growth chamber. Two weeks later, seedlings were planted in soil in plastic pots and transferred to a greenhouse under natural sunlight. For GA_3_ treatment, wild-type and *JcDREB2* overexpressing rice seeds were kept in water for 2 days for germination, then the seeds were incubated in Yoshida’s culture solution containing 10 μM GA_3_ for 10 days under natural sunlight. As an alternative GA treatment, plants grown in a greenhouse under natural sunlight were sprayed daily with 100 μM GA_3_ for 6 days (10-day-old seedlings) or 10 days (4-week-old seedlings). For the salt tolerance assay, 2-week-old seedlings were incubated in Yoshida’s culture solution containing 150 mM NaCl for 5 days, followed by incubation in Yoshida’s culture solution for 10 days at 25°C under 16/8 h (light/dark) conditions in a growth chamber, and then the survival rates were calculated. The relative electrolyte leakage (REL) and proline content were determined 2 days after salt treatment. Leaf samples were collected 2 days after salt treatment. All tests were repeated three times with three biological replicates for each test.

### Physiological Parameters, CAT and Superoxide Dismutase (SOD) Activity Measurements

About 0.2 g of leaf sample was used for each determination. Firstly, each sample was washed five times with deionized water and placed in a test tube containing 10 mL of deionized water. The leaf samples were immersed and vibrated continuously at 25°C for 2 h, then a conductivity meter was used to measure the electrical conductivity (B1) of the solution. Next the samples were boiled for 15 min, and after cooling the solutions to room temperature, their conductivity (B2) was measured again. The REL was calculated according to the following formula: REL (%) = B1/B2 × 100. The content of free proline in the rice leaves was determined as previously described (Bates et al., 1973). The content of malondialdehyde (MDA) in the leaves was determined as described by Cai et al. (2015). The activities of catalase (CAT) and superoxide dismutase (SOD) in the rice leaves were determined as previously described (Azooz et al., 2009).

### RNA Isolation and Expression Analysis

Total RNA was isolated from the leaves of 14-day-old rice seedlings using TRIzol reagent (Invitrogen, USA) according to the manufacturer’s instructions. Total RNA was extracted from roots, stem cortexes, flowers, and seeds of physic nut plants using a HiPure Plant RNA Mini kit (Promega, Madison, WI, USA) following the manufacturer’s instructions. First strand cDNA was obtained by reverse transcription of total RNA using M-MLV reverse transcriptase (Promega, Madison, WI, USA) following the manufacturer’s instructions. Primers used in the experiment are listed in Supplementary Table S1. Semi-quantitative RT-PCR with gene-specific primer pairs was used to detect the level of *JcDREB2* gene expression in wild-type and transgenic plants. The *OsUbiquitin* gene was used as a control.

Quantitative real-time PCR was performed in a reaction volume of 20 μL containing 2 μL of cDNA, 10 μL of 2 × SYBR Premix ExTaq, 0.4 μL forward primer (10 μmol), 0.4 μL reverse primer (10 μmol) and 7.2 μL of ddH2O using a LCS480 system (Roche^2^). Conditions for all quantitative real-time PCR amplifications were as follows: 95°C for 10 min for DNA polymerase activation, followed by 40 cycles of 95°C for 15 s and 60°C for 35 s. The relative expression level was calculated using the 2^-ΔΔCT^ method, and *OsUbiquitin* and *JcActin* were used as the reference genes for rice and physic nut, respectively. All experiments included three biological replicates, each with two technical replicates.

### Statistical Analysis

All experiments included three biological replicates, and the data collected were analyzed using the Duncan multiple range test (Duncan, 1955) with the SAS software package^3^.

## Results

### Isolation and Phylogenetic Analysis of *JcDREB2*

**FIGURE 1:**
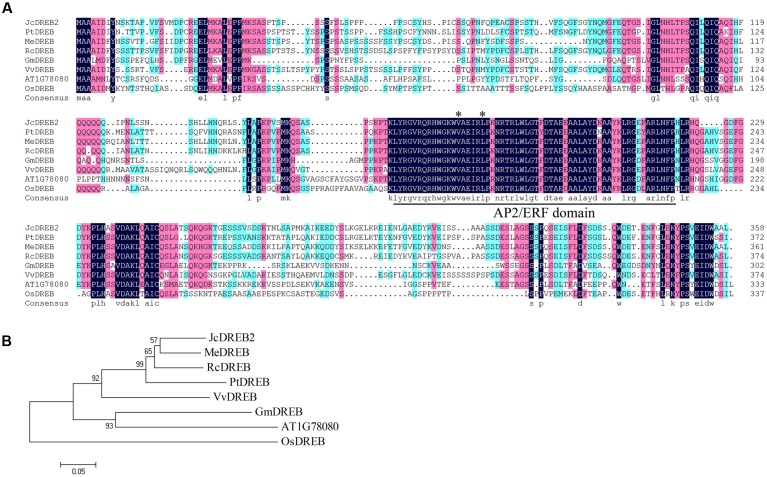
**Multiple sequence alignment and phylogenetic relationships of JcDREB2 homologs.** **(A)** The conserved DREB AP2/ERF domain is indicated as the underlined segment. *Stars* indicate the amino acid residues in the AP2/ERF domain reported to be conserved. **(B)** Phylogenetic relationships of JcDREB2 homologs constructed using MEGA 5 software. The accession numbers of the protein sequences retrieved from GenBank and species designations are as follows: Me, *Manihot esculenta* (OAY35862); Pt, *Populus trichocarpa* (XP_002307098); Rc, *Ricinus communis* (XP_002533146); Gm, *Glycine max* (XP_003526587); Vv, *Vitis vinifera* (XP_003635449); Os, *Oryza sativa* (XP_015625871).

The full-length *JcDREB2* cDNA was isolated from physic nut by reverse transcription PCR. The *JcDREB2* sequence so obtained contained an open reading frame of 1074 bp that encoded a protein of 358 amino acids with one conserved AP2/ERF domain. The *JcDREB2* sequence was deposited in the NCBI GenBank database under accession number JCGZ_24071. The conserved Val (14th) and Glu (19th) residues in the AP2/ERF domain are crucial in regulating the binding activity of DREB proteins to the DRE element (Cao et al., 2001). Further analysis of the deduced amino acid sequence indicated that the JcDREB2 protein contained these conserved 14th valine and 19th glutamic acid residues in the AP2/ERF domain and that it was highly homologous to DREB proteins from other plants (**Figure 1A**).

To further determine the relationship between JcDREB2 and other DREB proteins, a phylogenetic tree was established using the complete amino acid sequence (**Figure 1B**). Among the proteins included, the JcDREB2 protein was most closely related to cassava MeDREB, with a 75% match at the amino acid level (**Figures 1A,B**).

### Expression Patterns of *JcDREB2*

**FIGURE 2:**
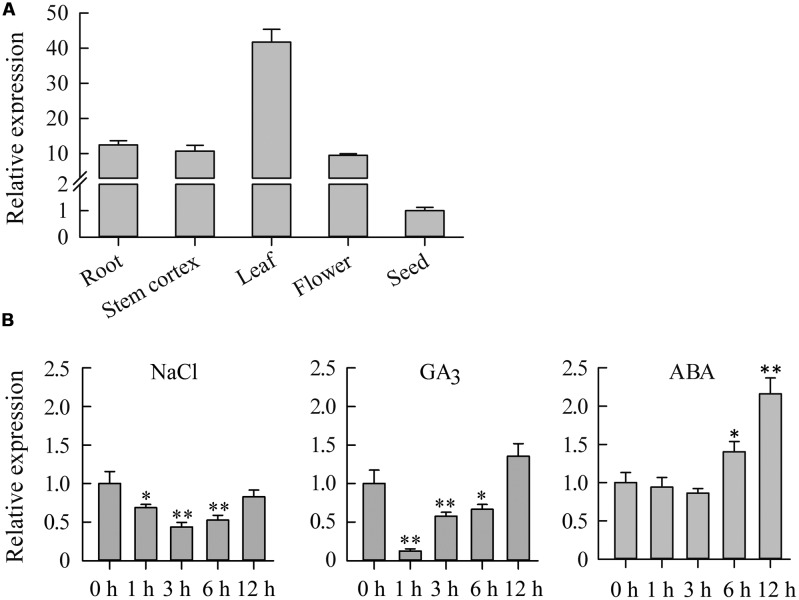
**Expression analysis of the *JcDREB2* gene in physic nut.** **(A)** qRT-PCR analysis of *JcDREB2* gene expression in roots, stem cortex, leaves, flowers, and seeds at 35 days after pollination. Relative expression was normalized with respect to the reference gene *JcActin* (internal control). Bars show means ± SD of three biological replicates. **(B)** qRT-PCR analysis of *JcDREB2* gene expression in leaves under different abiotic stresses. Bars show standard deviations of the replicates. Each assay was run in triplicate for three independent biological replicates. Values represent means of *n* = 3 ± SD (Duncan test: ^∗^*P* < 0.05, ^∗∗^*P* < 0.01).

To determine the expression pattern of *JcDREB2* in physic nut, we analyzed its expression in five different tissues: roots, stem cortexes, leaves, flowers, and seeds by real-time quantitative PCR (qRT-PCR) using *JcDREB2*-specific primers. *JcDREB2* was constitutively expressed in all of the tissues examined, but the highest level of expression was detected in leaves (**Figure 2A**).

Quantitative PCR analysis was performed to measure *JcDREB2* expression under various abiotic stresses, including salt, GA_3_ and ABA. As shown in **Figure 2B**, *JcDREB2* expression was repressed in physic nut leaves 1, 3, and 6 h after treatment with salt and GA_3_. However, under ABA stress, the level of *JcDREB2* transcript was markedly up-regulated from 6 to 12 h after treatment.

### JcDREB2 Is a Nuclear-Localized Protein

**FIGURE 3:**
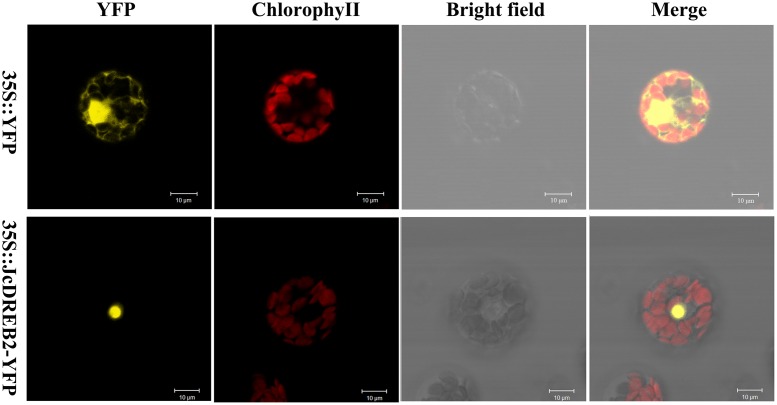
**Subcellular localization of *JcDREB2***.

In order to determine the subcellular localization of the JcDREB2 protein, we examined the localization of *JcDREB2*-YFP by laser scanning confocal microscopy (**Figure 3**). The YFP fluorescence produced by 35S::*JcDREB2*-YFP was localized in the nucleus, whereas YFP fluorescence produced by 35S::YFP was detected throughout the cell. These results indicate that JcDREB2 is a nuclear-localized protein.

### Overexpression of *JcDREB2* in Rice Produces a Severe Dwarf Phenotype

**FIGURE 4:**
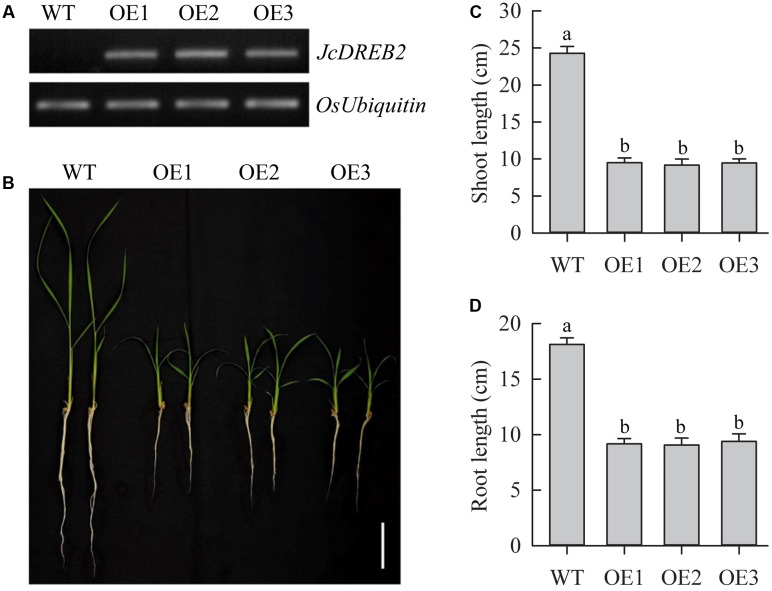
**Phenotype of *JcDREB2*-overexpressing rice plants.** **(A)** Relative levels of *JcDREB2* transcript in different transgenic rice plants (OE1, OE2, and OE3), determined by semi-quantitative RT-PCR; **(B)** Phenotype of wild-type and dwarf *JcDREB2*-overexpressing plants. Two-week-old seedlings cultured in water were photographed. Bar = 5 cm. **(C,D)** Relative shoot length **(C)** and root length **(D)** in wild-type plants and three *JcDREB2*-overexpressing lines after 14 days of growth on Yoshida’s culture solution. The experiment included three biological replicates. Values represent **(C,D)** means of *n* = 20 ± SD from three independent experiments and different letters above the columns indicate significant differences from the corresponding WT at the *p* < 0.01 level.

To investigate the function of *JcDREB2*, we overexpressed the *JcDREB2* gene in rice under the control of the CaMV 35S promoter. Changes in levels of *JcDREB2* transcripts in leaves were analyzed by semi-quantitative RT-PCR (**Figure 4A**). We observed that all transgenic rice plants overexpressing *JcDREB2* (*OeJcDREB2*) had a severe dwarf phenotype under normal growing conditions (**Figure 4B**). The shoot and root length was shorter in *OeJcDREB2* rice plants, compared with those of wild-type plants (**Figures 4C,D**).

### The Dwarf Phenotype of Transgenic Plants Is Rescued by the Application of Exogenous GA_3_

**FIGURE 5:**
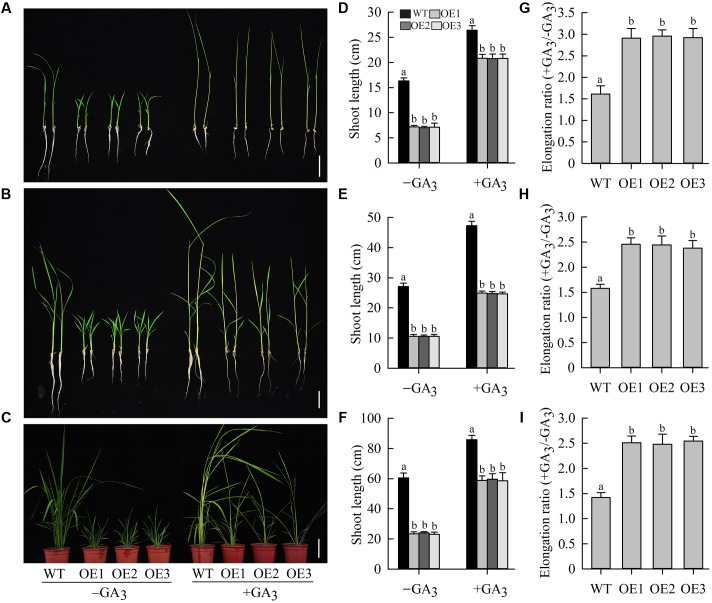
**Exogenous GA_3_ effectively reverses the GA-deficiency phenotype.** **(A)** Ten-day-old seedlings germinated and grown in Yoshida’s culture solution containing 10 μM GA_3_ or no GA_3_. Bar = 5 cm; **(B)** GA_3_ treatment. Ten-day-old seedlings were sprayed with 100 μM GA_3_ daily for 6 days. Bar = 5 cm. **(C)** GA_3_ treatment. Four-week-old seedlings were sprayed with 100 μM GA_3_ daily for 10 days. Bar = 10 cm. **(D)** Relative shoot length was measured on wild-type plants and three *JcDREB2*-overexpressing lines after 10 days of growth on Yoshida’s culture solution containing 10 μM GA_3_ or no GA_3_. **(E)** Elongation growth of 10-day-old *JcDREB2*-overexpressing and wild-type seedlings treated with GA_3_. Plant shoot length was measured 6 days after GA_3_ treatment. **(F)** Elongation growth of 4-week-old *JcDREB2*-overexpressing and wild-type seedlings treated with GA_3_. Plant shoot length was measured 10 days after GA_3_ treatment. **(G)** Elongation ratio (+GA_3_/-GA_3_) of 10-day-old seedlings germinated and grown in Yoshida’s culture solution. **(H)** Elongation ratio (+GA_3_/-GA_3_) of 10-day-old seedlings grown with GA_3_ or without GA_3_ for 6 days. **(I)** Elongation ratio (+GA_3_/-GA_3_) of 4-week-old *JcDREB2*-overexpressing and wild-type seedlings grown with GA_3_ or without GA_3_ for 10 days. The experiment included three biological replicates. Values represent **(D–I)** means of *n* = 15 ± SD from three independent experiments and different letters above the columns indicate significant differences from the corresponding WT at the *p* < 0.01 level.

Gibberellin, the most important of the hormones regulating shoot elongation, flowering, and seed development, plays an important role in determining plant height (Sakamoto et al., 2004). To examine whether the dwarf phenotype of *OeJcDREB2* rice plants was caused by GA deficiency, we cultivated wild-type and transgenic seedlings in Yoshida’s culture solution containing 10 μM GA_3_ for 10 days at the germination stage. Under these conditions, shoots of transgenic as well as of wild-type plants showed rapid elongation (**Figures 5A,D**), but the elongation ratio (+GA_3_/-GA_3_) of *OeJcDREB2* plants was significantly higher than that of the wild-type (**Figure 5G**). Furthermore, spraying *OeJcDREB2* rice plants with 100 μM GA_3_ rescued shoot elongation; in particular, the length of the leaf sheath was fully restored to a level similar to that in wild-type plants (**Figures 5B,C,E,F**). The elongation ratio (+GA_3_/-GA_3_) of *OeJcDREB2* plants was also significantly higher than that of the wild-type (**Figures 5H,I**). These results suggest that *OeJcDREB2* rice plants do indeed exhibit a classic reduced-GA phenotype.

### GA Biosynthesis Is Regulated by *JcDREB2* Expression

**FIGURE 6:**
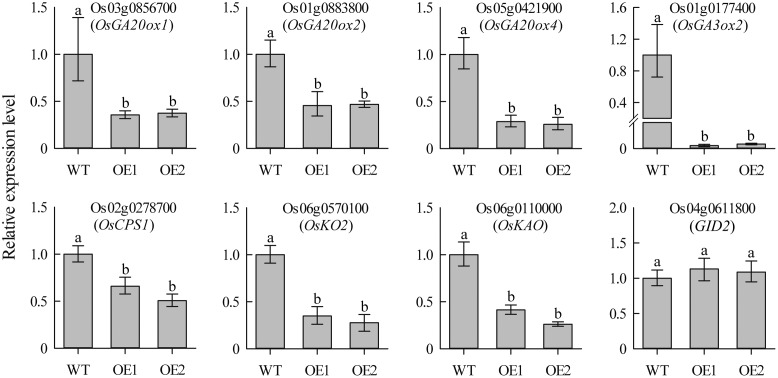
**Real-time PCR analysis of GA biosynthesis genes in leaves of 2-week-old seedlings.** Each PCR assay was run in duplicate for each of three independent biological replicates. Values represent means of *n* = 6 ± SD and different letters above the columns indicate significant differences from the corresponding WT at the *p* < 0.01 level.

To investigate whether overexpression of *JcDREB2* altered the expression of GA biosynthesis genes, we used qRT-PCR assays to detect the expression of genes encoding two key enzymes [gibberellin 20-oxidase (GA20ox) and gibberellin 3β-hydroxylase (GA3ox)] as well as other genes responsible for the early steps in GA biosynthesis, including *OsCPS1*, *OsKO2*, and *OsKAO*. As shown in **Figure 6**, the expression levels of *OsGA20ox1*, *OsGA20ox2*, *OsGA20ox4*, *OsGA3ox2, OsCPS1*, *OsKO2*, and *OsKAO* exhibited a significant reduction in *OeJcDREB2* seedlings compared with their levels in wild-type. GID2 (gibberellin-insensitive dwarf2) is involved in the GA signaling pathway in rice (Gomi et al., 2004). We therefore also examined the expression levels of the *GID2* gene, but no significant difference in *GID2* expression was seen between *OeJcDREB2* and wild-type plants (**Figure 6**).

### *JcDREB2* Negatively Regulates the Salinity-Induced Response in Rice

**FIGURE 7:**
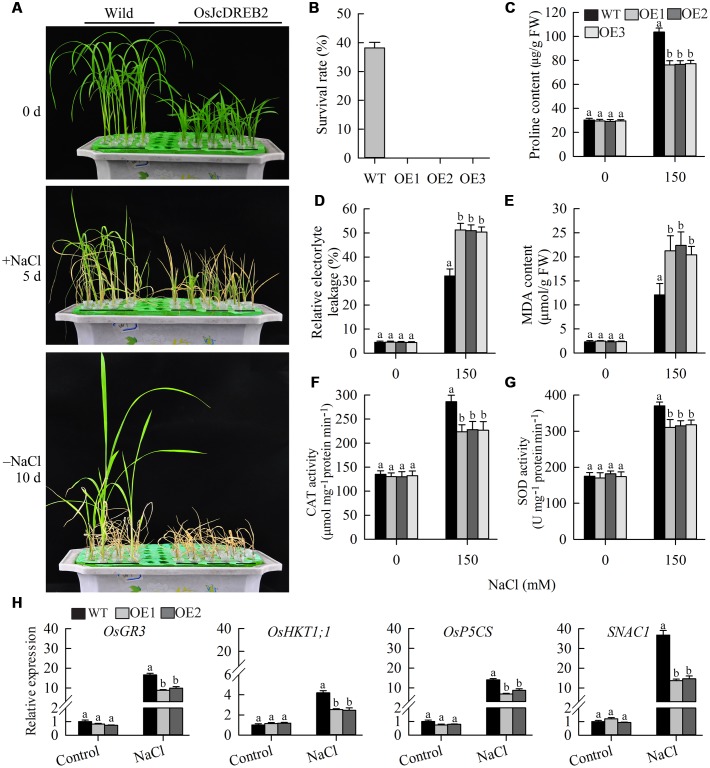
**Salt stress tolerance tests on *JcDREB2*-overexpressing rice lines and wild-type plants.** **(A)** Performance of wild-type and transgenic plants before and after salt treatment (150 mM NaCl), and after recovery for 10 days following salt treatment. The experiment included three biological replicates. **(B)** Survival rates of wild-type and transgenic plants after recovery for 10 days following salt treatment. **(C)** Proline content of leaves before and after salt treatment. **(D,E)** Relative electrolyte leakage (REL) **(D)** and MDA content **(E)** of leaves before and after salt treatment. Values represent means of *n* = 15 ± SD from three independent experiments and different letters above the columns indicate significant differences from the corresponding WT at the *p* < 0.01 level. **(F,G)** Activity of catalase (CAT) **(F)** and superoxide dismutase (SOD) **(G)** in leaves before and after salt treatment. Values represent means of *n* = 15 ± SD from three independent experiments and different letters above the columns indicate significant differences from the corresponding WT at the *p* < 0.05 level. **(H)** Relative expression levels of salt stress-responsive genes. The experiment included three biological replicates, each with two technical replicates. Values represent means of *n* = 6 ± SD and different letters above the columns indicate significant differences from the corresponding WT at the *p* < 0.01 level.

To examine whether *JcDREB2* plays a role in salt stress tolerance, we compared the salt tolerance of *OeJcDREB2* and wild-type plants at the vegetative growth stage. After 5 days of salt treatment with 150 mM NaCl, 73.3% of wild-type seedlings remained green, whereas *OeJcDREB2* seedlings showed severe leaf aging and rolling (**Figure 7A**). The survival rates of wild-type and *OeJcDREB2* seedlings after 10 days of recovery were statistically analyzed. Our data showed that approximately 38% of the wild-type seedlings survived, whereas no *OeJcDREB2* seedlings survived after the recovery period (**Figure 7B**). However, overexpressing *JcDREB2* had no significant effect on drought tolerance (data not shown) in rice. Proline accumulation is considered to be a plant adaptive response to high salinity and drought stresses (Xiong et al., 2012). Under normal growth conditions, the proline content did not differ between wild-type and *OeJcDREB2* plants. However, under salt treatment, the proline content of *OeJcDREB2* plants was significantly lower than that of the wild-type (**Figure 7C**). This finding indicated that salt stress was more damaging to the *OeJcDREB2* plants than to the wild-type plants. Electrolyte leakage is an important index of cell membrane damage in plant stress responses. The relative level of electrolyte leakage from wild-type leaves was lower than that from *OeJcDREB2* leaves after the salt stress treatments (**Figure 7D**).

Membranes abound in polyunsaturated fatty acids which serve as antioxidants by capturing reactive oxygen species, thereby protecting plants from being damaged under extreme conditions. This process generates MDA, a typical marker of the oxidation of polyunsaturated fatty acids. The MDA content of wild-type plants was lower than that of *OeJcDREB2* plants under salt treatment (**Figure 7E**). These results suggested that there was more cell membrane damage in *OeJcDREB2* leaf cells than in wild-type leaf cells under salt stress.

Superoxide dismutase is an antioxidant enzyme that catalyzes the conversion of the superoxide radical into hydrogen peroxide, and CAT further catalyzes the transformation of harmful hydrogen peroxide into harmless water, so protecting plants from damage by reactive oxygen species arising as a result of abiotic stress. We analyzed CAT and SOD activities under salinity stress and normal conditions. The results suggested that the activities of CAT and SOD from *OeJcDREB2* leaves were lower than those from wild-type leaves under salinity stress, whereas no significant difference was found under normal growth conditions (**Figures 7F,G**).

Finally, we measured the expression levels of several genes which have previously been shown to respond to salt stress in rice seedlings, since overexpressing these genes could improve the salinity stress tolerance of transgenic plants. The products of the *OsHKT1;1* gene are crucial for salt tolerance in rice through the exclusion of Na^+^ ions from sensitive cells (Ren et al., 2005). The *OsGR3* gene products are involved in scavenging reactive oxygen species (Wu et al., 2015). Increases in the levels of expression of *OsHKT1;1*, *OsP5CS* and *OsGR3* under salt treatment were strongly impaired in the leaves of two *OeJcDREB2* plants (**Figure 7H**). In addition, the expression level of the salt-stress-related transcription factor gene *SNAC1* (*STRESS-RESPONSIVE NAC 1*) (Hu et al., 2006) was significantly lower in *OeJcDREB2* leaves than in wild-type leaves under salinity stress (**Figure 7H**).

## Discussion

The AP2/ERF proteins constitute quite a large family, with 119 members, in physic nut (Tang et al., 2016). Although at least 5 physic nut AP2/ERF genes, *JcERF* (Tang et al., 2007), *JcDREB* (Tang et al., 2011), *JcERF1* (Yang et al., 2014), *JcERF2* (Wang et al., 2015), and *JcERF011* (Tang et al., 2016), have been shown to play significant roles in plant development and abiotic stress responses, the biological function of most *JcAP2/ERF* genes is not known. In this study, we show that *JcDREB2* is a stress-responsive AP2/ERF gene and that, when overexpressed in rice, it has significant effects on plant growth and salinity stress.

Our results demonstrated that overexpression of *JcDREB2* in rice produced plants that were dwarfed with retarded growth (**Figure 4B**). The dwarf phenotype of *OeJcDREB2* plants could be restored by the application of GA_3_ (**Figure 5**). This shows that the dwarf phenotype is the result of a deficiency of bioactive GAs in the transgenic rice. These results are consistent with previous studies on GA-deficiency mutants in a wide range of plants, including barley (Jia et al., 2009), Arabidopsis (Barboza et al., 2013), potato (Roumeliotis et al., 2013), maize (Chen et al., 2014), and rice (Oikawa et al., 2004; Qin et al., 2013).

We also found that overexpression of *JcDREB2* in rice restricted shoot growth by inhibiting the expression of *OsGA20ox1*, *OsGA20ox2*, *OsGA20ox4*, *OsGA3ox2, OsCPS1*, *OsKO2*, and *OsKAO*, which encode key enzymes in the GA biosynthetic pathway. There is accumulating evidence to indicate that targeting *GA20ox*, *GA3ox*, *CPS*, *KO2* and *KAO* genes results in decreased levels of bioactive GA and dwarf plants. *GA20ox* and *GA3ox* catalyze the last two steps in active GA biosynthesis, converting inactive forms of GA such as GA20 into active forms such as GA1 (Roumeliotis et al., 2013). Downregulating the expression of *GA20ox* and *GA3ox* causes severe dwarf phenotypes by altering levels of active GAs (Carrera et al., 2000; Itoh et al., 2004; Roumeliotis et al., 2013; Regnault et al., 2014). Similarly, the *CPS* gene, which encodes ent-copalyl diphosphate synthase, and mutated forms of the gene, have been isolated from various plants, and a typical phenotype in these mutants is dwarfism (Sakamoto et al., 2004). Ent-kaurenoic acid oxidase (KAO) catalyzes the conversion of ent-kaurenoic acid (KA) to gibberellin GA12, the precursor of all GAs (Regnault et al., 2014). The kao1 kao2 double mutant exhibits a typical GA-deficient dwarf phenotype (Regnault et al., 2014). Taken together, our results suggest that *JcDREB2* restricts shoot growth by downregulating the expression of key GA biosynthetic enzymes. GID2 is a positive regulator of GA signaling which is essential for the GA-mediated degradation of DELLA proteins such as SLR1 (slender Rice 1) (Gomi et al., 2004). Our study suggests that the expression of *GID2* is not significantly changed in *JcDREB2* overexpressers (**Figure 6**). This result further demonstrates that *JcDREB2* negatively regulates shoot growth processes by repressing GA biosynthesis and not via signal transduction.

A large number of studies have reported that many DREB gene products are involved in abiotic stress responses and that overexpression of *DREB* genes can enhance plant tolerance of abiotic stress (Lata and Prasad, 2011). In the present study, we found that the expression of *JcDREB2* in physic nut leaves was repressed by salinity stress (**Figure 2B**). Overexpressing the *JcDREB2* gene in rice reduced its tolerance to salt stress, leading to more pronounced leaf aging and rolling (**Figure 7A**). When plants suffer from drought and salinity stresses, there are rapid physiological changes in response to these adverse conditions (Seki et al., 2007). Consequently, physiological signs related to the plants’ responses to abiotic stress can be used as reference points in the evaluation of plant stress tolerance. Proline is considered to play an important part in defense mechanisms (Nanjo et al., 1999). A strong link between tolerance of abiotic stress and the accumulation of proline has been demonstrated by knocking out and overexpressing the *P5CS* gene in a number of plants (Nanjo et al., 1999; Bartels and Sunkar, 2007). Our study suggested that there was less accumulation of proline in the transgenic rice plants compared with the wild-type rice plants under salinity stress (**Figure 7C**), indicating that proline may be one factor responsible for the low tolerance shown by *JcDREB2* transgenic plants toward salinity stress. Furthermore, the leaves of the transgenic rice plants showed higher REL and MDA content compared with the leaves of wild-type rice (**Figures 7D,E**), showing that the wild-type plants were better able to resist the adverse effects of salinity stress. SOD and CAT play important roles in protecting plants against the toxic effects of reactive oxygen species produced as a result of various abiotic stresses (Azooz et al., 2009). Our study indicated that there was higher SOD and CAT activity in the wild-type rice plants compared with the transgenic rice plants under salinity stress (**Figures 7F,G**), which suggests that the transgenic rice plants were disadvantaged under conditions of high salinity. Since transcriptional regulatory networks responsive to salinity and drought stresses have been identified in rice and Arabidopsis, research into the expression of salinity stress-responsive genes in the networks could supply information crucial for dissecting the functions of *JcDREB2* in salinity stress tolerance. Our results indicated that the expression of stress-responsive genes such as *OsHKT1;1*, *OsP5CS*, *OsGR3*, and *SNAC1* was significantly lower in transgenic rice compared to wild-type under salinity stress (**Figure 7H**). Related research has shown that overexpressing these genes may improve the tolerance of transgenic plants to salinity stress (Ren et al., 2005; Hu et al., 2006; Wu et al., 2015). Our findings indicate that *JcDREB2* affected salt stress tolerance in the transgenic rice plants at least partially owing to decreases in the expression of stress-responsive genes under salinity stress.

## Conclusion

We characterized the physic nut AP2/ERF gene *JcDREB2*, the expression of which was repressed by salinity and GA_3_, but was induced by ABA. Overexpression of *JcDREB2* in rice resulted in dwarf and GA-deficient phenotypes, and these phenotypes were restored by exogenous GA_3_ treatment. Overexpression of *JcDREB2* enhanced sensitivity to salt stress in transgenic rice plants. These results increase our understanding of the roles played by this physic nut AP2/ERF transcription factor in plant growth and responses to abiotic stress.

## Author Contributions

The research was conceived and designed by YT and CL. The experiments were performed by YT, and the data were analyzed by YT, KL, JZ, XL, KX, YZ, JQ, DY, and JW. The manuscript was written and revised by YT and CL. All the authors read and approved the final manuscript.

## Conflict of Interest Statement

The authors declare that the research was conducted in the absence of any commercial or financial relationships that could be construed as a potential conflict of interest.

## Footnotes

1 http://www.ncbi.nlm.nih.gov/

2 http://www.roche.com/

3 http://www.sas.com/en_us/software/sas9.html
